# Tactile angle discriminability improvement: roles of training time intervals and different types of training tasks

**DOI:** 10.1152/jn.00161.2019

**Published:** 2019-08-28

**Authors:** Wu Wang, Jiajia Yang, Yinghua Yu, Qiong Wu, Jiabin Yu, Satoshi Takahashi, Yoshimichi Ejima, Jinglong Wu

**Affiliations:** ^1^Cognitive Neuroscience Laboratory, Graduate School of Natural Science and Technology, Okayama University, Okayama, Japan; ^2^Cognitive Neuroscience Laboratory, Graduate School of Interdisciplinary Science and Engineering in Health Systems, Okayama University, Okayama, Japan; ^3^Japan Society for the Promotion of Science, Tokyo, Japan; ^4^Section on Functional Imaging Methods, National Institute of Mental Health, Bethesda, Maryland; ^5^Beijing Institute of Technology, Beijing, China

**Keywords:** between-session learning, generalization, tactile angle discriminability, training time interval

## Abstract

Perceptual learning, which is not limited to sensory modalities such as vision and touch, emerges within a training session and between training sessions and is accompanied by the remodeling of neural connections in the cortex. However, limited knowledge exists regarding perceptual learning between training sessions. Although tactile studies have paid attention to between-session learning effects, there have been few studies asking fundamental questions regarding whether the time interval between training sessions affects tactile perceptual learning and generalization across tactile tasks. We investigated the effects of different training time intervals on the consecutive performance of a tactile angle discrimination (AD) task and a tactile orientation discrimination (OD) task training on tactile angle discriminability. The results indicated that in the short-interval training group, AD task performance significantly improved in the early stage of learning and nearly plateaued in the later stage, whereas in the long-interval training group, significant improvement was delayed and then also nearly plateaued in the later stage; additionally, improved OD task performance resulted in improved AD task performance. These findings suggest that training time interval affects the early stage of learning but not the later stage and that generalization occurs between different types of tactile tasks.

**NEW & NOTEWORTHY** Perceptual learning, which constitutes important foundations of complicated cognitive processes, is learning better perception skills. We demonstrate that training time interval can affect the early stage of learning but not the later stage. Moreover, a tactile orientation discrimination training task can also improve tactile angle discrimination performance. These findings may expand the characteristics of between-session learning and help understand the mechanism of the generalization across tactile tasks.

## INTRODUCTION

Perceptual learning is a process that improves sensory discriminability independently of sensory modalities through experience and practice performing specific sensory tasks, such as visual and haptic training (Ahissar and Hochstein 1997; Imai et al. 2003; Karni and Sagi 1993; Kurylo et al. 2017; Teodorescu et al. 2013; Trzcinski et al. 2016; Watanabe and Sasaki 2015; Wong et al. 2013). Recent studies in the visual and auditory domains have indicated that in terms of the learning improvement time course, two distinct learning stages exist: fast learning within a session and slow learning between sessions (Atienza et al. 2002; Karni and Sagi 1993; Molloy et al. 2012; Qu et al. 2010). Slow learning can last longer than fast learning once the skill is acquired (Qu et al. 2010); however, it can also be easily disrupted by events that occur after training during the consolidation phase of learning (Banai et al. 2010). Although recent tactile studies have focused on between-session learning effects (Imai et al. 2003; Trzcinski et al. 2016; Walter-Walsh et al. 2009; Weiss et al. 2007; Wong et al. 2013), few studies have focused on fundamental questions surrounding how the training time interval between sessions affects tactile perceptual learning and generalization across tactile tasks. This gap motivated us to investigate how training time intervals and different types of training tasks improve tactile object discriminability.

Learning between sessions, which follows and is distinct from learning within a session, is thought to be a consolidation process (Atienza et al. 2002; Molloy et al. 2012; Qu et al. 2010; Roth et al. 2005) that is immune to interference (Banai et al. 2010; Zach et al. 2005). Moreover, the effects of this process do not immediately appear after training but require at least 8 h after practice has ended (Atienza et al. 2002; Gais et al. 2000; Karni et al. 1992; Karni and Sagi 1993) and are accompanied by neural changes in the primary sensory cortices and/or other high-level areas (Atienza et al. 2002; Berry et al. 2010; Debowska et al. 2016; Qu et al. 2010; Siuda-Krzywicka et al. 2016). In particular, previous studies have shown that continuous exposure to a sensory stimulus can modify neural representations and neuronal responsiveness in primary sensory cortices (Dahmen and King 2007; Debowska et al. 2016; Shibata et al. 2014; Wang et al. 1995), a process that may also underlie between-session learning. However, this learning effect might decrease after several days or a week without continuous exposure (Aberg and Herzog 2012; Abraham 2003). In the touch domain, recent evidence indicates that perceptual learning performance in tactile multisession training linearly increases and then plateaus (Imai et al. 2003; Trzcinski et al. 2016; Walter-Walsh et al. 2009; Weiss et al. 2007), which might indicate skill consolidation and experience-dependent plasticity in the brain. Although skill learning certainly requires a period of time between training sessions, if the training time interval is too long, then between-session learning may disintegrate as a result of the lack of continuous exposure to the stimulus and the decay of memory traces. Our first aim is to address how 1-wk-interval training could impair between-session learning across multiple sessions relative to 1-day-interval training.

Furthermore, different types of training tasks that share similarities with an untrained task can also improve performance in the untrained task (Beatty et al. 2015; Berry et al. 2010; Ortiz and Wright 2009; Wang et al. 2016; Zhang et al. 2016, 2017). These training tasks may not only result in enhanced sensory processing (Atienza et al. 2002; Berry et al. 2010; Ortiz and Wright 2009; Watanabe and Sasaki 2015) but may also improve high-level cognitive processes, such as working memory (WM), prediction, and attention (Beatty et al. 2015; Siuda-Krzywicka et al. 2016; Spence and McGlone 2001; Wang et al. 2016; Zhang et al. 2016), and these improvements may generalize to untrained tasks and stimuli. For example, there are many aspects of WM processing, including maintenance, decision making, and updating, and training on specific aspects of WM (e.g., maintenance and updating) that are functionally shared by a trained and a target task leads to generalization (Beatty et al. 2015; Zhang et al. 2016). Although generalization across tasks has been shown in the tactile domain as a result of sensory processing and/or cognition (Grant et al. 2000; Spengler et al. 1997; Trzcinski et al. 2016), the mechanism of the generalization between different types of tactile tasks still remains unclear. Therefore, our second aim is to explore how a training task with shared stimulus features and task procedures can improve performance in another task.

To address the questions mentioned above, we applied the tactile angle discrimination (AD) task used in our previous studies (Wu et al. 2010; Yang et al. 2014; Yu et al. 2018). The AD task measures spatial perception of touch involving advanced cognition, such as WM and attention. In the first experiment, we aimed to explore the effects of training time interval (1 day vs. 1 wk) on tactile perceptual learning across sessions. Thus two subject groups were assigned to different time interval training regimes (i.e., 1-day vs. 1-wk groups) to consecutively perform five sessions of the AD task. By comparing the AD threshold changes in these two training regimes across sessions, we further assessed the disintegration of the between-session learning effect in the long-interval training regime. In the second experiment, to compare the learning effects that stem from different types of training tasks, we added a new subject group that was instead trained using the tactile orientation discrimination (OD) task, but the pre- and posttest assessments still used the AD task. Furthermore, a subject group that only underwent the pre- and posttest using the AD task was recruited as a control group to verify the learning effects of both the first and second experiments.

## MATERIALS AND METHODS

### Experiment 1: Training Time Interval Effects

#### Subjects.

Twenty healthy volunteer undergraduate and graduate students were recruited to participate in this experiment. Subjects were randomly and equally allocated into two experimental groups: the 1-day-interval group (aged 22–30 yr, mean 26.3 ± 2.62 yr; 8 men), with a 1-day-interval training regime, and the 1-wk-interval group (aged 22–29 yr, mean 25.1 ± 3.07 yr; 8 men), with a 1-wk-interval training regime. All subjects were right-hand dominant, and we confirmed that their index fingers were free of injuries and calluses. Each group received training in five consecutive sessions in the AD task, but the time interval between the sessions was different across the groups. In the 1-day-interval group, for the subjects’ personal reasons, three subjects received two sessions of training in 1 day, but the time interval between these two sessions exceeded 8 h, which is the minimum amount of time required to consolidate memory (Atienza et al. 2002; Gais et al. 2000; Karni et al. 1992; Karni and Sagi 1993); there was also one subject for whom one period between sessions was 2 days. Therefore, the mean time interval between sessions was 0.96 ± 0.13 days. In the 1-week-interval group, the time span between the sessions of nine subjects was 1 wk, whereas that of one subject was 10 days for the period between the fourth session and the fifth session, as a result of travel obligations. Therefore, the mean time interval between sessions was 7.06 ± 0.19 days. All subjects provided written informed consent in compliance with the policies of the local medical ethics committee of Okayama University. The testing procedures were reviewed and approved by the local medical ethics committee of Okayama University.

#### Tactile AD task.

##### apparatus and stimuli.

**Fig. 1.**
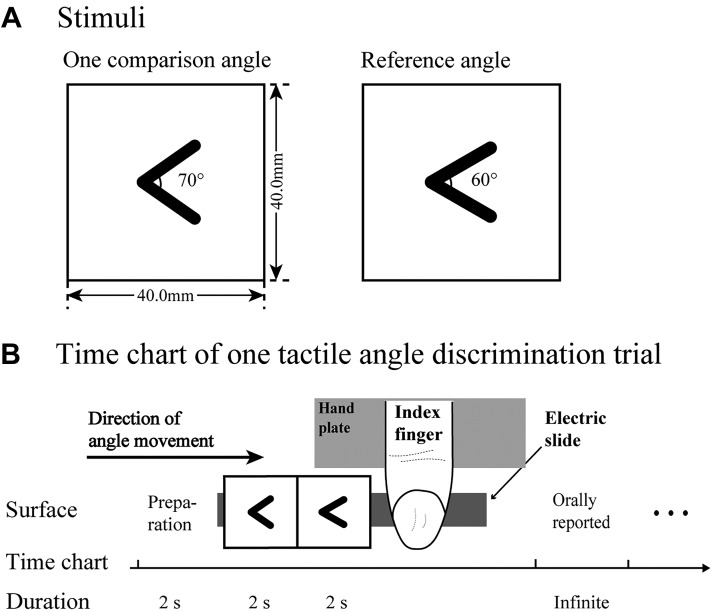
Example of angle stimuli and 1 trial of the tactile angle discrimination (AD) task. *A*: an example of the reference angle (60°) and 1 (70°) of 10 comparison angles used in *experiment 1*. *B*: 1 trial of the tactile AD task. First, the subject’s right index finger was gently placed onto the plate, remaining fixed and static during each trial of the task. Angles in *A* were clamped on the apparatus and were horizontally moved by an electric slide, as shown by the arrow. Only when the angles passed under the subject’s index finger pad could he or she perceive the angle. Subjects were instructed to orally report which angle was larger. If they could not identify which angle was larger, they could indicate that the angles were the same.

We used two-dimensional (2-D) raised angles that had been employed in previous studies (Wu et al. 2010; Yang et al. 2014; Yu et al. 2018). These angles were composed of plastic lines (8.0 mm long, 1.5 mm wide, and 1.0 mm high) and plastic square bases (40.0 mm long and wide, 3.0 mm high). Figure 1*A* shows an illustration of a pair of angles. All types of 2-D plane angles could be made by symmetrically changing the spatial dimensions of two raised lines along an imaginary bisector at the center of this square base. To minimize the impact of the end-point distance on angle discrimination, we employed one reference angle (60°, 8.0-mm end-point distance) and 10 comparison angles that differed from the reference angle by ±2°, ±4°, ±6°, ±8°, and ±10° and had end-point distances that were 7.8 and 8.2 mm, 7.5 and 8.5 mm, 7.3 and 8.7 mm, 7.0 and 8.9, and 6.8 and 9.2 mm; these angles were measured to an accuracy of ±0.2°. During *experiment 1*, each angle in a pair of angles that included the reference angle and a comparison angle was presented in succession to the index finger pad of the subject. The apparatus including an electric slide was applied to allow raised angles to slide passively across the finger. The angles were held horizontally on the apparatus, and the right hand of the subject was fixed with nylon tape to the fixed plate to maintain passive touching (Fig. 1*B*). Throughout the entire experiment, only the index finger could contact the angle stimulus.

##### procedure.

**Fig. 2.**
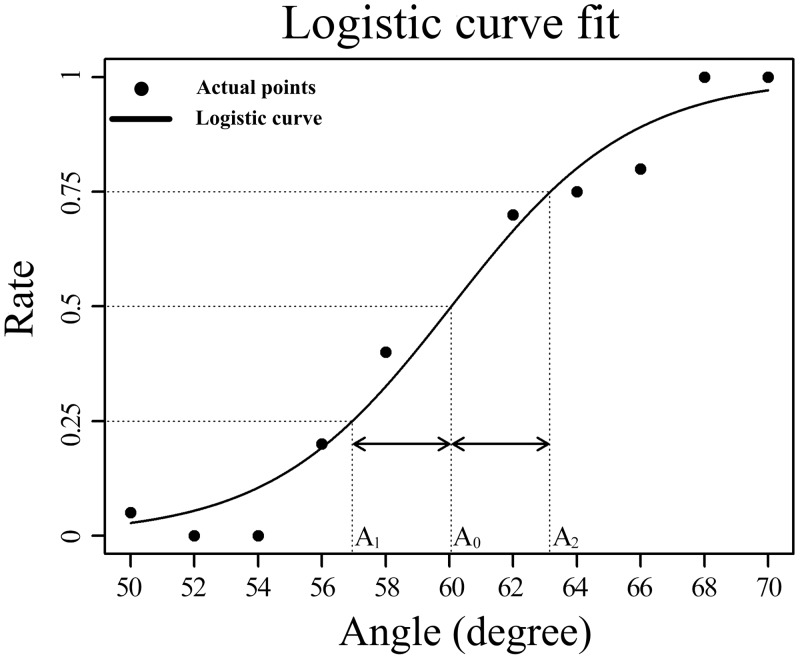
Logistic curve fit. The accuracy data of the subject in the angle discrimination (AD) task were applied to this logistic function (1). To cater to the logistic curve used to calculate the AD threshold, the subject’s responses to the AD task need to be transferred to a frequency distribution (black circles). Thereby, the rates in the condition of the reference angles > comparison angle (left side of the reference angle) are folded from top to bottom by being reduced by 1. Black circles represent the revised rates of 1 subject (H.B.O.) who performed 1 session of the AD task. Solid line represents the logistic curve acquired by the least-squares method when the residual was the smallest. Dashed lines indicate 2 points (*A_1_*; 0.25) and (*A_2_*; 0.75), and the discrimination threshold was (*A_2_* − *A_1_*)/2.

The subjects were blindfolded and seated at a table with the apparatus. To maintain the index finger and arm in the same straight position, the subjects’ right hands were attached to the plate, and their forearms were fixed to a device that was perpendicular to the electric slide. The experimenter subsequently instructed the subjects to lightly place the right index fingers at the terminal point of the hand plate (Fig. 1B). We defined the AD threshold as being half the angular distance between the 25% and 75% probability intersection points (Fig. 2). Next, according to the individual’s ability to judge the relative sizes of the reference angle and the comparison angle, the AD thresholds were calculated. A pair of angles was subsequently carried by the slide to slide passively across the index finger pad so that the subject could perceive the sizes of the angles and orally report which of the two angles was larger. Also, we kept the movement speed of the slide unchanged at 20.0 mm/s. Because the distance between the reference angle and the comparison angle was 31.8 ± 0.8 mm, the interstimulus time interval between these two angles was ~1.6 s. A pseudorandom order for presenting pairs of angles was applied in which the reference angle emerged in either the first or second position of each pair, but this information was never provided to the subjects. Before the experiment, each subject experienced at least 10 practice trials with other angles to become familiar with the experimental procedure. Each pair of angles used in the formal experiment subsequently emerged 10 times in a pseudorandom order. To avoid uncomfortable sensations on the index finger, enforced 3-min breaks occurred after each series of 20 trials. Thus each session consisted of 100 trials and lasted ~40 min.

#### Data processing and analysis.

In this study, a logistic curve was used as an approximate curve to estimate AD thresholds (Fig. 2). The logistic curve has been extensively used in psychophysical experiments to measure thresholds (Hoehler 1995; Kuehn et al. 2017; Weder et al. 1998), and its equation is as follows:

(1) $$y=\frac{1}{1+e^{\alpha +\beta x}}.$$

In this equation, α and β are two parameters of the equation; β represents the logistic growth, and *−*α/β is representative of the *x*-value of the sigmoid curve midpoint.

Before performing the analysis of variance, we ensured that the data (see supplemental dataset for *experiment 1*; All Supplemental Material is available at https://doi.org/10.6084/m9.figshare.7824719) were normally distributed by applying a one-sample Kolmogorov–Smirnov (K-S) test and checking the Q-Q diagram for further confirmation that the data were basically near the straight line. Because each subject was repeatedly measured five times in this discrimination task, which might result in a subject random effect, the *lmer* function in R language was used to conduct repeated-measures ANOVA and control this random effect. Moreover, the *lsmeans* function in R language was used for the post hoc contrast.

### Experiment 2: Effects of Different Types of Tactile Training Tasks

#### Subjects.

Twenty volunteers who did not participate in *experiment 1* were recruited for *experiment 2*. In this experiment, we randomly and equally assigned subjects to the experimental group (aged 22–30 yr, mean 24.2 ± 2.62 yr; 7 men; all right-hand dominant) or the control group (aged 22–33 yr, mean 26.6 ± 3.6 yr; 6 men; 1 left-hand dominant) and obtained consent from subjects to participate in *experiment 2* using the same criteria as in *experiment 1*. The experimental group received the pre- and posttest of the AD task and three sessions of tactile orientation discrimination training, whereas the control group only received the pre- and posttest of the AD task. The time interval between the pre- and posttest was 3 days, during which the experimental group received one session of training each day. For personal reasons, one subject from the experimental group received two sessions of training on the third day, but the time interval between the two training sessions exceeded 8 h. Therefore, the experimental group’s mean period between the pretest and posttest was 2.9 ± 0.32 days.

#### Tactile OD task.

##### apparatus and stimuli.

**Fig. 3.**
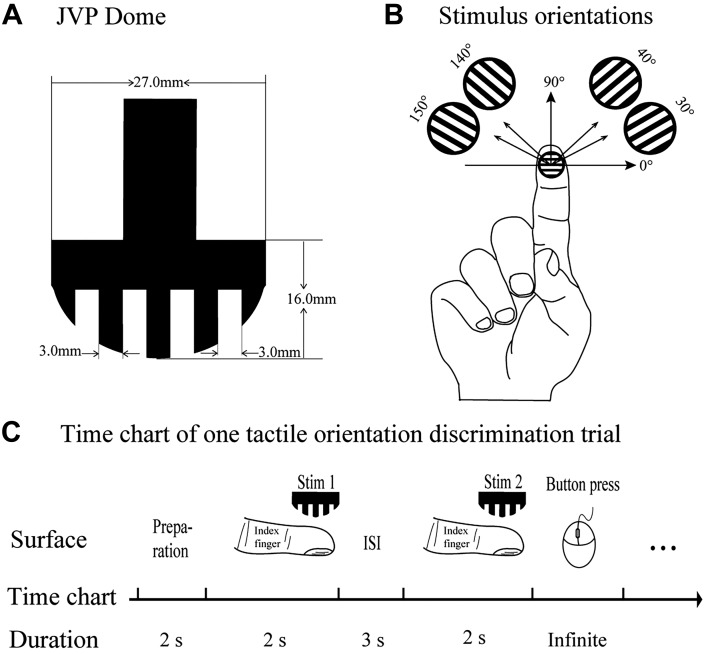
Tactile orientation stimuli and an example of 1 trial of the tactile orientation discrimination (OD) task. *A*: a Johnson-Van Boven-Phillips (JVP) dome was used to manually present tactile orientations in a tactile OD task. *B*: 4 different tactile orientations were presented in this task. *C*: 1 trial of a tactile OD task. First, the subject’s right index finger was fixed in the holder to keep the finger immobile and in a supinated position. A pair of grating orientations was then manually presented to the distal part of the index finger in succession; the subject’s objective was to determine whether the orientations of the 2 gratings (*Stim 1* and *Stim 2*) were the same or different. Throughout the entire experiment, only the index finger pad could contact the dome. ISI, interstimulus interval.

We used a rounded plastic Johnson-Van Boven-Phillips (JVP) dome that had been employed in our previous study (Yu et al. 2013) to present tactile orientations (Fig. 3*A*). This dome was cut into square-wave gratings with an equal ridge and gap width (3 mm). In this discrimination task, four different tactile orientations were presented to the distal part of the right index finger (Fig. 3*B*). To avoid common orientations (e.g., horizontal and vertical) that could be easily and semantically coded, we chose relatively uncommon grating orientations (30°, 40°, 140°, and 150°), thereby forcing the subjects to form their perceptual representations in the brain. These orientations comprised four sets of the same orientation pairs (e.g., 30° and 30°) and six sets of different orientation pairs (e.g., 30° and 140°).

We developed a manual device to operate the presentation of the orientations. The device consisted of two parts: the fixation part for the hand and the tactile stimulus presentation part. During this experiment, the device was placed on the table. The fixation part kept the hand stable and the index finger pad upright. The tactile stimulus presentation part consisted of a rolling unit and a shaft unit. The rolling unit was responsible for controlling the grating orientations, and the shaft unit made the grating dome move up and down in a straight line. In each trial, the force by which the grating dome contacted the finger pad remained constant and was restricted to 10 N. To better help the experimenter manually present the stimuli, we developed a program to prompt the operator to control the grating orientation and presentation time. This program was written using E-prime (version 1.0; Psychology Software Tools).

##### procedure.

Blindfolded subjects sat comfortably at a table with their right hand supinated in a fixation apparatus to keep the hand and finger immobile. To avoid any possible hints from the mechanical sound of the dome rotation, we placed headphones on the subject’s ears and played meaningless white noise. For each trial, a pair of grating orientations was manually presented to the distal part of the index finger in succession, each lasting 2 s, with a 3-s interstimulus interval (Fig. 3*C*), and the force by which the dome pressed the finger pad remained constant. The subject’s objective was to determine whether the orientations of the two gratings were the same or different. Each pair of grating orientations was presented 10 times in a pseudorandom order to mix the two conditions (same and different orientation pairs) with 100 trials per training session. Before the experiment, each subject participated in at least 10 practice trials using other orientations to familiarize themselves with the experimental procedure. It was necessary to provide a 3-min break after each series of 20 trials, and a complete session lasted ~45 min. Finally, accuracy was used to estimate task performance.

## RESULTS

### Experiment 1

**Fig. 4.**
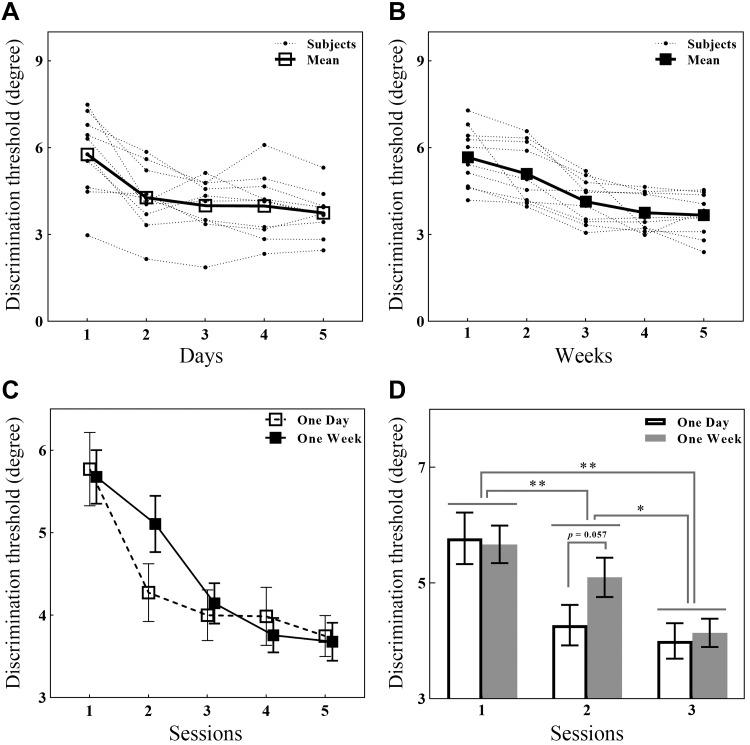
Effects of different training time intervals on the threshold in the tactile angle discrimination (AD) task. *A*: individual (dots) and group average (open squares) AD threshold performance in the 1-day-interval group across 5 sessions. AD threshold sharply decreased in *session 2* and then plateaued in *sessions 2–5*. *B*: individual (dots) and group average (closed squares) AD threshold performances in the 1-wk-interval group across 5 sessions. AD threshold remained nearly unchanged in *sessions 1* and *2*, sharply decreased in *session 3*, and then plateaued in *sessions 3–5*. *C*: changes in AD threshold performances in 2 different time-interval groups with consecutive trainings. Note that lower thresholds indicate better performance. *D*: to better show and compare the difference between AD threshold changes in the 2 groups, AD thresholds in the first 3 sessions are plotted as a histogram. Values are means ± SE. *P < 0.05; **P < 0.01.

To estimate the extent of perceptual improvement in the 1-day-interval group vs. the 1-wk-interval group, we performed a 2 (interval: 1 day and 1 wk) × 5 (training session: initial to final) repeated-measures ANOVA with the AD threshold as the dependent measure. Because sex may have possibly affected tactile spatial acuity, it was added as a covariate to the variance analysis. We observed a significant main effect of training session (*F*_4, 80_ = 50.20, *P* < 0.001) and an nonsignificant effect of sex (*F*_1, 20_ = 0.024, *P* = 0.87), and we also found a significant interval × training session interaction effect (*F*_4, 80_ = 3.31, *P* = 0.015; Fig. 4, *C* and *D*). Specifically, the post hoc comparison [Tukey’s honestly significant difference (HSD) on a double-tailed *t* test] indicated that the AD threshold sharply decreased in *session 2* (*t*_80_ = 6.47, *P* < 0.001) and then plateaued in *sessions 2–5* in the 1-day-interval group (Fig. 4*A*), whereas the AD threshold remained nearly unchanged in *sessions 1* and *2* (*t*_80_ = 2.46, *P* = 0.11), sharply decreased in *session 3* (*t*_80_ = 6.60, *P* < 0.001, *t*_80_ = 4.14, *P* < 0.001), and then plateaued in *sessions 3–5* in the 1-wk-interval group (Fig. 4*B*). Interestingly, the difference between the AD thresholds in *session 2* for the two training regimes was marginally significant (*t*_34.13_ = 1.97, *P* = 0.057; Fig. 4*D*). No other significant effects were observed.

**Fig. 5.**
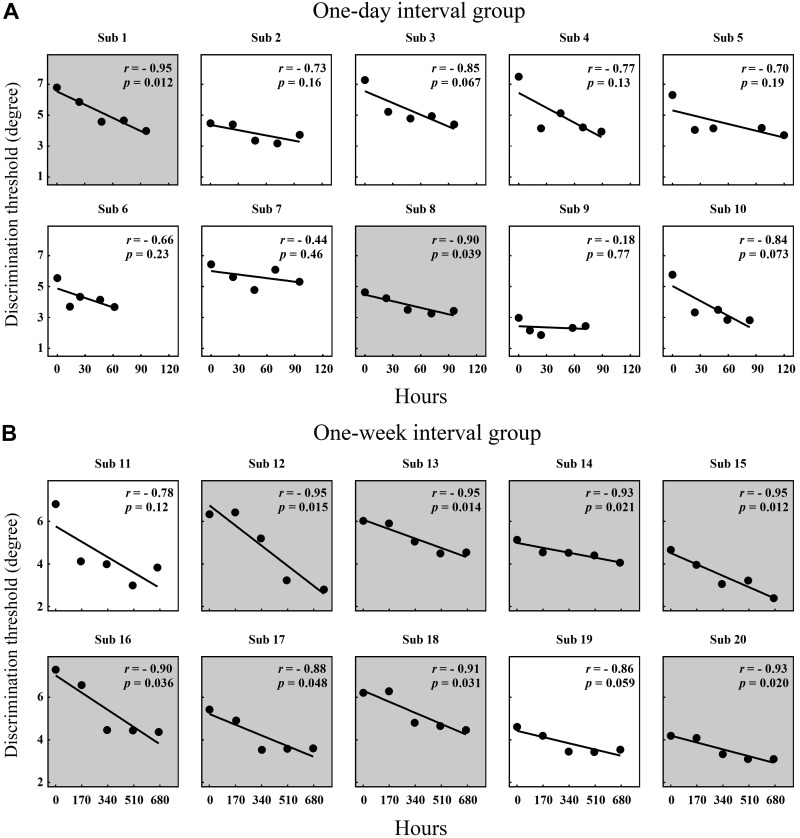
Generalized linear models that predict the angle discrimination (AD) thresholds with actual hours between sessions. These models are based on data from the AD thresholds in 5 sessions for each subject of 2 groups (1 day vs. 1 wk) with their corresponding actual hours between sessions. *A*: in the 1-day-interval group, only 2 (*sub 1* and *8*) of 10 linear models had significant linear fits. Regression lines are shown. *B*: in contrast, a significant linear fit was observed in 8 of 10 linear models (except *sub 11* and *19*) in the 1-wk-interval group. Regression lines are shown. Light gray backgrounds indicate that the linear model has a significant linear fit (*P* < 0.05).

To further explore the characteristics of tactile perceptual learning on different time-interval groups, we run a linear regression using SPSS (SPSS Statistics, version 22.0; IBM, Armonk, NY) for each subject of two groups (1 day vs. 1 wk) with the AD thresholds in five sessions as a function of actual hours elapsed between sessions (Fig. 5). The results show that 8 of 10 linear regressions had significant linear fits (*P* < 0.05) in the 1-wk-interval group (Fig. 5*B*); in contrast, a significant linear fit was only observed in 2 of 10 linear regressions in the 1-day-interval group (Fig. 5*A*). Plausibly, training effects in the 1-wk group were better captured by a 1/session function, which might suggest two different learning functions across different time-interval training regimes.

### Experiment 2

**Fig. 6.**
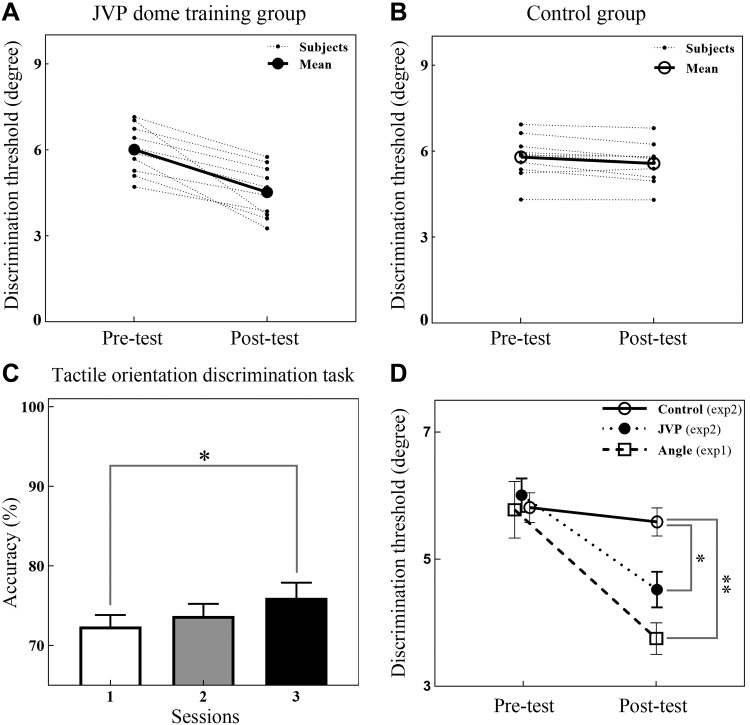
Effects of different types of training tasks on angle discrimination (AD) ability. *A*: individual (dots) and group average (closed circles) AD threshold performance during the pre- and posttest in the Johnson-Van Boven-Phillips (JVP) dome training group. *B*: individual (dots) and group average (open circles) AD threshold performance during the pre- and posttest in the control group. *C*: tactile orientation discrimination task performance during 3 training sessions; performance in *session 3* significantly improved. *D*: comparison of AD threshold improvements across the 3 groups; pretest scores in the 3 groups were nearly equal, whereas posttest scores were significantly different. Improvement in the angle training group was best, and that in the JVP training group was better than that in the control group. Values are means ± SE. *P < 0.05; **P < 0.01.

The analysis tools were the same as those used in *experiment 1*, using the K-S test to ensure that data (see supplemental datasets for *experiment 2*; All Supplemental Material is available at https://doi.org/10.6084/m9.figshare.7824719) were normally distributed and using the *lmer* and *lsmeans* functions in R language for the analysis of variance. First, the training effect of the tactile OD task was estimated via one-way repeated-measures ANOVA, which indicated that the session effect was significant (*F*_2, 20_ = 3.91, *P* = 0.037). The post hoc comparison (Tukey’s HSD) further indicated that the accuracy in *session 3* was significantly higher than that in *session 1* (*t*_20_ = 2.76, *P* = 0.031), and the other comparisons were not significant (Fig. 6*C*). These findings indicated that tactile orientation discrimination performance was remarkably better in *session 3*, although the means of the accuracy measures gradually increased across the three sessions. To better compare the learning effects resulting from the different training tasks, the data for the 1-day-interval group from *sessions 1* and *5* of *experiment 1* were combined with the data from *experiment 2* and analyzed. Therefore, we ran a 2 (testing: pretest and posttest) × 3 (training regime: angle vs. JVP dome vs. control) repeated-measures ANOVA with the AD threshold as the dependent measure, and sex was added as a covariate to the analysis. We observed a significant main effect of testing (*F*_1, 30_ = 86.33, *P* < 0.001; Fig. 6*D*) and an nonsignificant effect of sex (*F*_1, 30_ = 0.029, *P* = 0.87). Importantly, we also found a significant testing × training regime interaction effect (*F*_2, 30_ = 15.83, *P* < 0.001; Fig. 6*D*). A simple interaction analysis (Tukey’s HSD) indicated that the posttest scores were lower than the pretest scores in the angle and JVP training regimes (*t*_30_ = 8.73, *P* < 0.001; *t*_30_ = 6.40, *P* < 0.001; Fig. 6, *A* and *D*), whereas the difference between the post- and pretest scores for the control group was not significant (*t*_30_ = 0.97, *P* = 0.34; Fig. 6, *B* and *D*); moreover, the pretest scores of the three groups were nearly equal, whereas the posttest scores for the angle and JVP training regimes were lower than those in the posttest for the control group (*t*_41.89_ = 4.57, *P* < 0.001; *t*_42.16_ = 2.65, *P* = 0.030; Fig. 6*D*).

**Fig. 7.**
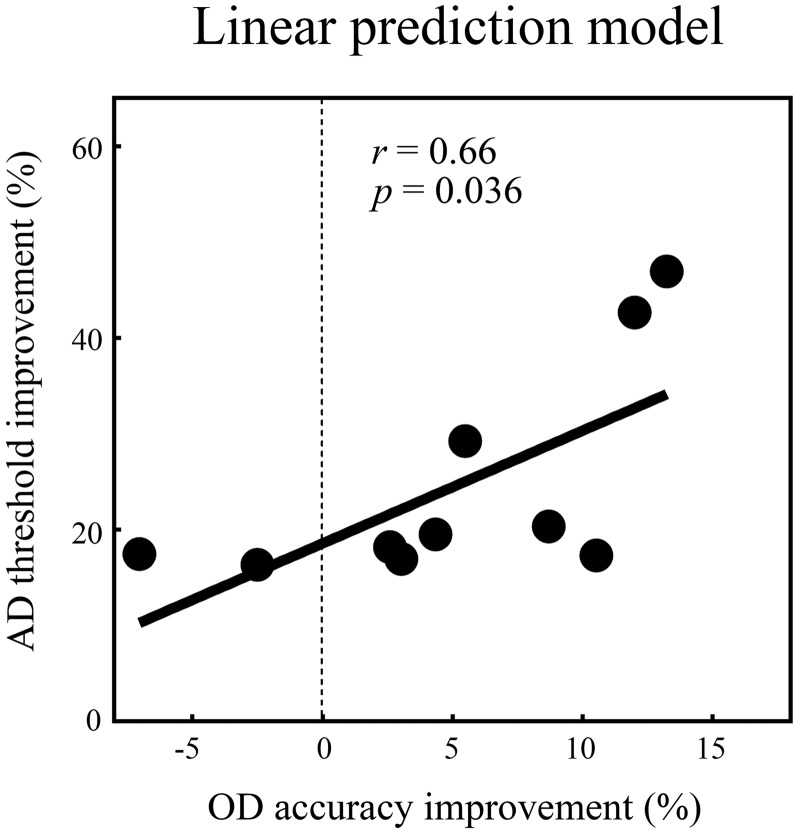
Linear prediction model. Orientation discrimination (OD) accuracy improvement (%) is defined as (3rd accuracy − 1st accuracy)/1st accuracy in the tactile OD training task; AD threshold improvement (%) is defined as (pretest − posttest)/pretest in the AD task. The OD accuracy improvement rate predicted the AD threshold improvement rate with a significant fit (*P* < 0.05), and we noted that the intercept of the model differed significantly from 0 (*P* = 0.001; 95% confidence interval: 10.04 to 27.06).

To further determine whether subjects with better learning rates [(3rd accuracy − 1st accuracy)/1st accuracy] in the tactile OD training task also showed a higher learning rate [(pretest − posttest)/pretest] in the AD task, we run a linear regression using SPSS (SPSS Statistics, version 22.0; IBM) with the AD threshold improvement (%) as a function of the accuracy improvement (%). The result showed that the accuracy improvement rate indeed predicted the AD threshold improvement rate with a significant fit (*P* = 0. 036; Fig. 7), and the intercept of the model differed significantly from zero (*P* = 0.001; 95% confidence interval: 10.04 to 27.06), which might indicate that the AD threshold improvement benefited from not only the OD accuracy improvement but also other factors such as passive touching.

## DISCUSSION

### Experiment 1

We assessed how perceptual learning would be interactively affected by different training time intervals between sessions and multisession training. Our main finding is that short-interval training significantly improved AD ability in the early stage of learning, whereas long-interval training delayed its improvement by one session in the same stage of learning. Moreover, once emerged in the later stage, the learning effect was not affected by the training time interval. We suggest that the training time interval can affect the early stage of learning but not the later stage. Furthermore, the marginal difference between the second sessions in the two training regimes may further suggest that between-session learning disintegrates during the early consolidation of a skill. These findings may be an interesting addition to the characteristics of between-session learning, except for its prominent improvement in the early stage (Karni and Sagi 1993; Molloy et al. 2012).

We found that the AD ability in the 1-day-interval group improved after training (Imai et al. 2003; Trzcinski et al. 2016; Walter-Walsh et al. 2009; Weiss et al. 2007) (Fig. 4, *A* and *C*). One straightforward explanation for this result is that the skill required during acquisition might be well consolidated in the period between sessions. Furthermore, continuous exposure to tactile angles may also increase the neural sensitivity of the primary somatosensory cortex (SI) or facilitate other neural functions endemic to the SI. Although we cannot accurately determine the neural processing of the brain during acquisition, such as by coding stimulus features (Banai et al. 2010; Seitz et al. 2005), building a decision network (Jacobs 2009; Lu et al. 2010), or integrating practice trials to a learning threshold (Little et al. 2017), skill consolidation may involve time-dependent changes to the brain (Gais et al. 2000; Karni et al. 1992; McGaugh 2000). After 1 day of consolidation, the AD skill acquired in the first session was remarkably demonstrated in the second session, which might suggest that the acquired skills had been carefully processed and were being continually stabilized in long-term memory (Banai et al. 2010; McGaugh 2000) following modifications in neural representations and neuronal responsiveness (Abraham 2003; Debowska et al. 2016; Wang et al. 1995).

In the 1-wk-interval group, we found that AD ability improvement did not appear in the second session but did appear in the third session (Fig. 4, *B* and *C*), which may indicate that early between-session learning disintegrated in 1 wk, although it did not disappear completely. This effect could have occurred because the early memory trace acquired during acquisition may have decayed over 1 wk (Hardt et al. 2013), and one more session of training may reinforce the remaining learning memory trace and facilitate the retention of the acquired skills (Dayan et al. 2014). Furthermore, the effect might not benefit from the neural sensitivity of the SI, which might have worked in several days in the 1-day-interval training regime, which might provide an additional mechanistic explanation for the learning difference between different time-interval training regimes (Fig. 5, *A* and *B*). Therefore, we further speculate that early between-session learning may depend on the maintenance of memory traces (McGaugh 2000). Because daily tactile experiences are characterized as random and uncertain, it is difficult to influence between-session learning in this way (Banai et al. 2010; Seitz et al. 2005). Hence, we believe that interference might not be the main factor that causes a meaningful loss of acquired tactile learning (Hardt et al. 2013).

### Experiment 2

We assessed whether the OD training task could improve AD ability and how angle discriminability could benefit from a different type of training task. Our results revealed that AD ability could be improved by training not only with the AD task but also with the OD task (Fig. 6*D*) and that training effects in the OD task could proportionally scale across the AD ability improvement (Fig. 7). We suggest that the improvement in OD ability can generalize to AD ability (Fig. 6, *A* and *C*). This finding may contribute to understanding the mechanism of generalization across tactile tasks.

Improvement in AD ability with the OD training task may occur because the two tasks share features that involve common primary sensory processing and/or high-level cognition abilities. In some sense, the linear prediction model with a significant intercept might indicate that the two aspects had worked together, although the current evidence was somewhat inadequate. Specifically, since short-term perceptual training can induce modifications in the primary sensory cortex (Atienza et al. 2002; Berry et al. 2010; Debowska et al. 2016; Qu et al. 2010), similar or shared perceptual features may be easily represented and processed in the primary sensory cortex (Foffani et al. 2008; Ortiz and Wright 2009). Therefore, we contend that the OD training task might improve representations in SI for tactile spatial acuity, which may result in generalization to the AD task. Moreover, training involving high-level cognitive processes, such as WM, prediction, and attention, might also lead to generalization (Wang et al. 2016). For example, training on specific aspects of WM could improve performance in untrained tasks that functionally share these cognitive processes (Beatty et al. 2015; Dahlin et al. 2008; Salminen et al. 2012; Zhang et al. 2016). Because OD and AD tasks require the performance of similar processing procedures (e.g., encoding, maintaining, and decision making), the OD training task might improve the ability to maintain individual representations of encoded stimuli, which may be generalized to AD task performance. Likewise, one study also found generalization across tactile tasks indicating that temporal order discrimination training could transfer to temporal discrimination of other tactile stimuli (Trzcinski et al. 2016), which probably results from procedural or cognitive learning (Spengler et al. 1997; Trzcinski et al. 2016).

### General Discussion

In the present study, we found that the training time interval can affect early learning between sessions, which supplements and enriches the understanding of between-session learning; moreover, later learning between sessions is immune to changes in the training time interval, which provides new evidence that perceptual learning can be preserved over a long time period. In addition, perceptual learning can emerge not only from training on the current task but also by training on other tasks with similarities that are shared with the current task. Shared similarities involving primary processing and/or advanced cognition processes may underlie generalization across tactile tasks. Therefore, these findings may support the understanding that short-term training sessions promote between-session learning and that training on fundamental and shared perceptual skills may enable extensive perceptual learning.

We found that the difference between AD ability improvements that resulted from AD task training and those that stemmed from OD task training was not significant (Fig. 6*D*); however, the improvement that resulted from the same task training was better. One possibility is that the behaviors necessary to perform these tasks may share advanced cognitive processes (e.g., WM) as a result of the similarities in the processing procedure and number of trials, and their essential differences may exist in their primary processing. Because the tactile angle is composed of two different orientation lines (Wu et al. 2010), compared with tactile angle processing, tactile orientation processing may be substantially easier and more fundamental within SI. Furthermore, the reverse hierarchy theory postulates that simple stimulus learning matches the spatial generalization of higher sensory areas (Ahissar and Hochstein 1997, 2004); thus tactile orientation identification may benefit tactile angle identification, which may facilitate stimulus encoding in WM and lead to generalization. However, tactile orientation processing cannot completely replace tactile angle processing. Therefore, the improvement resulting from training on the same task would be better. Additionally, one study found generalization not between tasks but between fingers (Sathian and Zangaladze 1997). A possible reason for not finding generalization between tasks is the use of a fixed standard stimulus in a haptic grating discrimination task, which could easily form a single stimulus representation and eliminate considerable WM involvement (e.g., memory updating) (Zhang et al. 2016). However, generalization between fingers may depend predominantly on mediation from regions outside SI (e.g., SII) (Imai et al. 2003; Sathian and Zangaladze 1997).

Although many possible factors may result in AD ability improvement, the present study indicated that more specific capabilities, such as tactile spatial acuity and WM, were responsible. Our study design, however, could not completely distinguish which of these two learning effects resulted from the improvement in perception and cognition; therefore, we have discussed both possible reasons. Further study needs to control one factor and discuss the learning effect that stems from the other factor. These separate confirmations may provide a better understanding of the mechanism of generalization across tactile tasks. Additionally, our findings may be confounded by engagement in other tasks of our laboratory that might affect perception and cognition abilities, although this effect may be small. Thus further study should also avoid confusion related to engaging in other tasks as much as possible.

## GRANTS

This work was supported by Japan Society for the Promotion of Science KAKENHI Grants JP17J40084, JP18K15339, JP18H05009, JP18H01411, JP18K18835, and JP17K18855.

## DISCLOSURES

No conflicts of interest, financial or otherwise, are declared by the authors.

## AUTHOR CONTRIBUTIONS

W.W., J. Yang, Y.Y., Q.W., S.T., Y.E., and J.W. conceived and designed research; W.W. and J. Yu performed experiments; W.W., J. Yang, and J. Yu analyzed data; W.W., J. Yang, and Y.Y. interpreted results of experiments; W.W. prepared figures; W.W. and J. Yang drafted manuscript; W.W. and J. Yang edited and revised manuscript; W.W., J. Yang, Y.Y., Q.W., S.T., and Y.E. approved final version of manuscript.

## References

[B1] Aberg KC, Herzog MH About similar characteristics of visual perceptual learning and LTP. Vision Res 61: 100–106, 2012. doi:10.1016/j.visres.2011.12.013. 22289647

[B2] Abraham WC How long will long-term potentiation last? Philos Trans R Soc Lond B Biol Sci 358: 735–744, 2003. doi:10.1098/rstb.2002.1222. 12740120 PMC1693170

[B3] Ahissar M, Hochstein S Task difficulty and the specificity of perceptual learning. Nature 387: 401–406, 1997. doi:10.1038/387401a0. 9163425

[B4] Ahissar M, Hochstein S The reverse hierarchy theory of visual perceptual learning. Trends Cogn Sci 8: 457–464, 2004. doi:10.1016/j.tics.2004.08.011. 15450510

[B5] Atienza M, Cantero JL, Dominguez-Marin E The time course of neural changes underlying auditory perceptual learning. Learn Mem 9: 138–150, 2002. doi:10.1101/lm.46502. 12075002 PMC182592

[B6] Banai K, Ortiz JA, Oppenheimer JD, Wright BA Learning two things at once: differential constraints on the acquisition and consolidation of perceptual learning. Neuroscience 165: 436–444, 2010. doi:10.1016/j.neuroscience.2009.10.060. 19883735 PMC2797123

[B7] Beatty EL, Jobidon ME, Bouak F, Nakashima A, Smith I, Lam Q, Blackler K, Cheung B, Vartanian O Transfer of training from one working memory task to another: behavioural and neural evidence. Front Syst Neurosci 9: 86, 2015. doi:10.3389/fnsys.2015.00086. 26082694 PMC4451342

[B8] Berry AS, Zanto TP, Clapp WC, Hardy JL, Delahunt PB, Mahncke HW, Gazzaley A The influence of perceptual training on working memory in older adults. PLoS One 5: e11537, 2010. doi:10.1371/journal.pone.0011537. 20644719 PMC2904363

[B9] Dahlin E, Neely AS, Larsson A, Bäckman L, Nyberg L Transfer of learning after updating training mediated by the striatum. Science 320: 1510–1512, 2008. doi:10.1126/science.1155466. 18556560

[B10] Dahmen JC, King AJ Learning to hear: plasticity of auditory cortical processing. Curr Opin Neurobiol 17: 456–464, 2007. doi:10.1016/j.conb.2007.07.004. 17714932

[B11] Dayan E, Averbeck BB, Richmond BJ, Cohen LG Stochastic reinforcement benefits skill acquisition. Learn Mem 21: 140–142, 2014. doi:10.1101/lm.032417.113. 24532838 PMC3929848

[B12] Debowska W, Wolak T, Nowicka A, Kozak A, Szwed M, Kossut M Functional and structural neuroplasticity induced by short-term tactile training based on Braille reading. Front Neurosci 10: 460, 2016. doi:10.3389/fnins.2016.00460. 27790087 PMC5061995

[B13] Foffani G, Chapin JK, Moxon KA Computational role of large receptive fields in the primary somatosensory cortex. J Neurophysiol 100: 268–280, 2008. doi:10.1152/jn.01015.2007. 18400959 PMC2493497

[B14] Gais S, Plihal W, Wagner U, Born J Early sleep triggers memory for early visual discrimination skills. Nat Neurosci 3: 1335–1339, 2000. doi:10.1038/81881. 11100156

[B15] Grant AC, Thiagarajah MC, Sathian K Tactile perception in blind Braille readers: a psychophysical study of acuity and hyperacuity using gratings and dot patterns. Percept Psychophys 62: 301–312, 2000. doi:10.3758/BF03205550. 10723209

[B16] Hardt O, Nader K, Nadel L Decay happens: the role of active forgetting in memory. Trends Cogn Sci 17: 111–120, 2013. doi:10.1016/j.tics.2013.01.001. 23369831

[B17] Hoehler FK Logistic equations in the analysis of S-shaped curves. Comput Biol Med 25: 367–371, 1995. doi:10.1016/0010-4825(95)00013-T. 7554853

[B18] Imai T, Kamping S, Breitenstein C, Pantev C, Lütkenhöner B, Knecht S Learning of tactile frequency discrimination in humans. Hum Brain Mapp 18: 260–271, 2003. doi:10.1002/hbm.10083. 12632464 PMC6871959

[B19] Jacobs RA Adaptive precision pooling of model neuron activities predicts the efficiency of human visual learning. J Vis 9: 22, 2009. doi:10.1167/9.4.22. 19757931

[B20] Karni A, Sagi D The time course of learning a visual skill. Nature 365: 250–252, 1993. doi:10.1038/365250a0. 8371779

[B21] Karni A, Tanne D, Askenasy JJ, Sagi D No dreams, no memory: the effect of REM sleep deprivation on learning a new perceptual skill. Soc Neurosci Abstr 18: 387, 1992.

[B22] Kuehn E, Doehler J, Pleger B The influence of vision on tactile Hebbian learning. Sci Rep 7: 9069, 2017. doi:10.1038/s41598-017-09181-6. 28831156 PMC5567334

[B23] Kurylo DD, Waxman R, Kidron R, Silverstein SM Visual training improves perceptual grouping based on basic stimulus features. Atten Percept Psychophys 79: 2098–2107, 2017. doi:10.3758/s13414-017-1368-8. 28681180

[B24] Little DF, Zhang YX, Wright BA Disruption of perceptual learning by a brief practice break. Curr Biol 27: 3699–3705.e3, 2017. doi:10.1016/j.cub.2017.10.032. 29174894 PMC5848209

[B25] Lu ZL, Liu J, Dosher BA Modeling mechanisms of perceptual learning with augmented Hebbian re-weighting. Vision Res 50: 375–390, 2010. doi:10.1016/j.visres.2009.08.027. 19732786 PMC2824067

[B26] McGaugh JL Memory—a century of consolidation. Science 287: 248–251, 2000. doi:10.1126/science.287.5451.248. 10634773

[B27] Molloy K, Moore DR, Sohoglu E, Amitay S Less is more: latent learning is maximized by shorter training sessions in auditory perceptual learning. PLoS One 7: e36929, 2012. doi:10.1371/journal.pone.0036929. 22606309 PMC3351401

[B28] Ortiz JA, Wright BA Contributions of procedure and stimulus learning to early, rapid perceptual improvements. J Exp Psychol Hum Percept Perform 35: 188–194, 2009. doi:10.1037/a0013161. 19170481 PMC2866737

[B29] Qu Z, Song Y, Ding Y ERP evidence for distinct mechanisms of fast and slow visual perceptual learning. Neuropsychologia 48: 1869–1874, 2010. doi:10.1016/j.neuropsychologia.2010.01.008. 20080117

[B30] Roth DA, Kishon-Rabin L, Hildesheimer M, Karni A A latent consolidation phase in auditory identification learning: time in the awake state is sufficient. Learn Mem 12: 159–164, 2005. doi:10.1101/87505. 15805314 PMC1074334

[B31] Salminen T, Strobach T, Schubert T On the impacts of working memory training on executive functioning. Front Hum Neurosci 6: 166, 2012. doi:10.3389/fnhum.2012.00166. 22685428 PMC3368385

[B32] Sathian K, Zangaladze A Tactile learning is task specific but transfers between fingers. Percept Psychophys 59: 119–128, 1997. doi:10.3758/BF03206854. 9038414

[B33] Seitz AR, Yamagishi N, Werner B, Goda N, Kawato M, Watanabe T Task-specific disruption of perceptual learning. Proc Natl Acad Sci USA 102: 14895–14900, 2005. doi:10.1073/pnas.0505765102. 16203984 PMC1253567

[B34] Shibata K, Sagi D, Watanabe T Two-stage model in perceptual learning: toward a unified theory. Ann N Y Acad Sci 1316: 18–28, 2014. doi:10.1111/nyas.12419. 24758723 PMC4103699

[B35] Siuda-Krzywicka K, Bola Ł, Paplińska M, Sumera E, Jednoróg K, Marchewka A, Śliwińska MW, Amedi A, Szwed M Massive cortical reorganization in sighted Braille readers. eLife 5: e10762, 2016. doi:10.7554/eLife.10762. 26976813 PMC4805536

[B36] Spence C, McGlone FP Reflexive spatial orienting of tactile attention. Exp Brain Res 141: 324–330, 2001. doi:10.1007/s002210100883. 11715076

[B37] Spengler F, Roberts TP, Poeppel D, Byl N, Wang X, Rowley HA, Merzenich MM Learning transfer and neuronal plasticity in humans trained in tactile discrimination. Neurosci Lett 232: 151–154, 1997. doi:10.1016/S0304-3940(97)00602-2. 9310302

[B38] Teodorescu K, Bouchigny S, Korman M Training haptic stiffness discrimination: time course of learning with or without visual information and knowledge of results. Hum Factors 55: 830–840, 2013. doi:10.1177/0018720812472503. 23964421

[B39] Trzcinski NK, Gomez-Ramirez M, Hsiao SS Functional consequences of experience-dependent plasticity on tactile perception following perceptual learning. Eur J Neurosci 44: 2375–2386, 2016. doi:10.1111/ejn.13343. 27422224 PMC5028271

[B40] Walter-Walsh K, Weiss T, Spohn D, Torma F, Miltner WH Spatial discrimination learning of electrocutaneous stimuli is influenced by the type of stimulation. Brain Res 1281: 47–57, 2009. doi:10.1016/j.brainres.2009.05.055. 19497308

[B41] Wang R, Wang J, Zhang JY, Xie X, Yang YX, Luo SH, Yu C, Li W Perceptual learning at a conceptual level. J Neurosci 36: 2238–2246, 2016. doi:10.1523/JNEUROSCI.2732-15.2016. 26888933 PMC6602041

[B42] Wang X, Merzenich MM, Sameshima K, Jenkins WM Remodelling of hand representation in adult cortex determined by timing of tactile stimulation. Nature 378: 71–75, 1995. doi:10.1038/378071a0. 7477291

[B43] Watanabe T, Sasaki Y Perceptual learning: toward a comprehensive theory. Annu Rev Psychol 66: 197–221, 2015. doi:10.1146/annurev-psych-010814-015214. 25251494 PMC4286445

[B44] Weder B, Nienhusmeier M, Keel A, Leenders KL, Ludin HP Somatosensory discrimination of shape: prediction of success in normal volunteers and parkinsonian patients. Exp Brain Res 120: 104–108, 1998. doi:10.1007/s002210050382. 9628408

[B45] Weiss T, Walter K, Spohn D, Richter M, Torma F, Miltner WH Spatial discrimination learning of electrocutaneous stimuli. Neurosci Lett 427: 83–87, 2007. doi:10.1016/j.neulet.2007.09.021. 17931775

[B46] Wong M, Peters RM, Goldreich D A physical constraint on perceptual learning: tactile spatial acuity improves with training to a limit set by finger size. J Neurosci 33: 9345–9352, 2013. doi:10.1523/JNEUROSCI.0514-13.2013. 23719803 PMC6618562

[B47] Wu J, Yang J, Ogasa T Raised-angle discrimination under passive finger movement. Perception 39: 993–1006, 2010. doi:10.1068/p6264. 20842975

[B48] Yang J, Yu Y, Kunita A, Huang Q, Wu J, Sawamoto N, Fukuyama H Tactile priming modulates the activation of the fronto-parietal circuit during tactile angle match and non-match processing: an fMRI study. Front Hum Neurosci 8: 926, 2014. doi:10.3389/fnhum.2014.00926. 25566010 PMC4266023

[B49] Yu Y, Yang J, Ejima Y, Fukuyama H, Wu J Asymmetric functional connectivity of the contra- and ipsilateral secondary somatosensory cortex during tactile object recognition. Front Hum Neurosci 11: 662, 2018. doi:10.3389/fnhum.2017.00662. 29416506 PMC5787555

[B50] Yu Y, Yang J, Wu J Limited persistence of tactile working memory resources during delay- dependent grating orientation discrimination. Neurosci Biomed Eng 1: 65–72, 2013. doi:10.2174/2213385211301010011.

[B51] Zach N, Kanarek N, Inbar D, Grinvald Y, Milestein T, Vaadia E Segregation between acquisition and long-term memory in sensorimotor learning. Eur J Neurosci 22: 2357–2362, 2005. doi:10.1111/j.1460-9568.2005.04415.x. 16262674

[B52] Zhang YX, Moore DR, Guiraud J, Molloy K, Yan TT, Amitay S Auditory discrimination learning: role of working memory. PLoS One 11: e0147320, 2016. doi:10.1371/journal.pone.0147320. 26799068 PMC4723131

[B53] Zhang YX, Tang DL, Moore DR, Amitay S Supramodal enhancement of auditory perceptual and cognitive learning by video game playing. Front Psychol 8: 1086, 2017. doi:10.3389/fpsyg.2017.01086. 28701989 PMC5487488

